# A randomized controlled trial to evaluate the efficacy of metacognitive training for older adults with depression (MCT-Silver) in Portugal: study protocol

**DOI:** 10.3389/fpsyg.2023.1167860

**Published:** 2023-10-31

**Authors:** Lara Guedes de Pinho, Celso Silva, César Fonseca, Bruno Morgado, Manuel Lopes, Steffen Moritz, Lena Jelinek, Brooke C. Schneider

**Affiliations:** ^1^Department of Nursing, University of Évora, Évora, Portugal; ^2^Comprehensive Health Research Centre (CHRC), Évora, Portugal; ^3^Higher School of Health, Polytechnic Institute of Beja, Beja, Portugal; ^4^Universitat Rovira i Virgili, Tarragona, Spain; ^5^Hospital Garcia de Orta, Almada, Portugal; ^6^University Medical Center Hamburg-Eppendorf, Hamburg, Germany

**Keywords:** depression, depressive symptoms, older adults, metacognitive training, intervention, cognitive behavioral therapy, randomized controlled trial, geriatric depression

## Abstract

**Introduction:**

Depression is one of the most common psychological disorders in later life. Although psychological interventions are recommended by treatment guidelines, most older adults with depression remain untreated. The aim of this study is to evaluate the efficacy of the Portuguese version of Metacognitive Training for Depression in later life (MCT-Silver).

**Methods:**

This is a study protocol of an observer-blind, parallel-group, randomized controlled trial to compare the efficacy of MCT-Silver with a treatment as usual (TAU) control group among older adults (age 65 years and older) with depressive symptoms according to the Montgomery-Asberg Depression Scale. Participants will be tested at three assessment time points (baseline, immediately following the intervention [8 weeks], and 3 months after the intervention). The primary outcome is change in self-rated depression symptoms assessed by the Beck Depression Inventory (BDI-II). Secondary outcomes include clinician-rated depression, self-esteem, dysfunctional beliefs, metacognitive beliefs, ruminations, attitudes toward aging and quality of life. A self-designed subjective appraisal rating scale consisting of 21-items will be used to assess participant acceptance of MCT-Silver.

**Discussion:**

MCT-Silver is an innovative intervention, which aims to reduce dysfunctional thoughts as well as depression-related behaviors and coping strategies through the metacognitive perspective. Until now, the training has only been tested in Germany. It is expected that after 8 weeks of treatment and 3 months later, the experimental group will demonstrate significant reductions in depressive symptoms, metacognitive beliefs, dysfunctional attitudes and ruminative responses compared to the TAU group. Moreover, quality of life, self-esteem, and attitudes towards aging will be significantly improved in MCT-Silver compared to the TAU group.

**Clinical trial registration:**

ClinicalTrials.gov, NCT05640492.

## 1. Introduction

According to the World Health Organization, depression is a common mental disorder, with around 280 million people diagnosed with the condition worldwide (World Health Organization, 2021). In 2019, depression was estimated to affect around 10.4% of adults, reaching about 5.7% in people over 60 years old (World Health Organization, 2021). In addition, depression is one of the main causes of years lived with disability (James et al., 2018; Santomauro et al., 2021) and it has been identified as a cause of dementia in older adults (Wu et al., 2020).

Depression is a mental disorder that can be manifested by persistent sadness, hopelessness, pessimism, loss of pleasure or interest in activities, and depressed mood (e.g., sad, irritable, empty). This symptomatology is present during most of the day, almost every day, for at least 2 weeks and most episodes can last considerably longer (American Psychiatric Association, 2014). Over time, depression can lead to cognitive and social dysfunction. It is therefore important to intervene as early as possible in order to minimize or eliminate disabilities and improve quality of life (Pinho et al., 2021).

The treatment of depression with antidepressant monotherapy is still a reality due to limited resources for treatment in community health centers or psychosocial rehabilitation facilities, but is limiteddue to side effects of medications, polypharmacy and poor adherence (e.g., due to memory impairment or organizational deficits). Given that rate of relapse after discontinuation of antidepressant medication reaches 56% (Kishi et al., 2023), implementation of psychotherapeutic strategies in the development of care management plans for depression is necessary. Moreover, the pharmaceutical treatment of depression is insufficient for the full rehabilitation of patients (Carvalho Pereira de Melo and Pereira de Melo, 2021; Mommaerts, 2022).

Metacognitive training for older adults with depression (MCT-Silver; Schneider et al., 2018; Bücker, 2019), is a cognitive-behavioral (CBT)-based group intervention, specifically designed for adults ages 60 years and older (all materials are available for free^1^). MCT-Silver aims to reduce the treatment gap by improving access to mental health care for the estimated 73% of older adults with depression who go untreated (Horackova et al., 2019). MCT-Silver is based on metacognitive training for psychosis (MCT; Moritz and Woodward, 2007) and metacognitive training for depression (D-MCT; Jelinek et al., 2015). Randomized controlled trials on D-MCT provide evidence for its efficacy on depressive symptoms (Jelinek et al., 2016), as well as metacognitive and negative cognitive beliefs (Hauschildt et al., 2022; Özgüç and Tanriverdi, 2022) at short- and intermediate follow-up intervals. Long-term efficacy of D-MCT remains equivocal (Jelinek et al., 2019). A pilot study examined the feasibility, acceptance, and effects of D-MCT as an add-on intervention in a group of older adults with depression who were completing an intensive inpatient treatment program (55+ years; *N* = 116). Per protocol analyses (*n* = 55) yielded a significant decrease in depressive symptoms (*d* = 1.06) and cognitive biases (*d* = 0.33). These results support the feasibility and acceptance of D-MCT among older adults with depression. A recently completed RCT in Germany, which compared MCT-Silver to an active control group (cognitive remediation; (Schneider et al., 2018)) yielded large and significant reductions on the primary outcome (Hamilton Depression Rating Scale) from baseline to post and 3-month follow-up (*d_MCT-Silver_* = 1.25 – 1.42; *d*_CR_ = 1.05 – 1.12). Group differences; however, did not reach significance (η_p_^2^ = 0.001 – 0.002). MCT-Silver yielded significant moderate to large effects compared to CR immediately following the intervention and after 3 months for self-reported depression (BDI-II: η_p_^2^ = 0.075 – 0.135) and rumination (RRS: η_p_^2^ = 0.087 – 0.127). A significant moderate effect was found for positive metacognitive beliefs (MCQ-PB) at post-assessment (η_p_^2^ = 0.067), but group differences did not reach significance at follow-up (η_p_^2^ = 0.027). Small reductions in negative cognitive beliefs (Dysfunctional Attitudes Scale-18B) at post- and at 3-month follow-up (*d_MCT-Silver_* = 0.24 – 0.30) were found for the MCT-Silver group, whereas negative cognitive beliefs were unchanged in the CR group (*d_CR_* = –0.06 – 0.06).

Like D-MCT, the main goal of MCT-Silver is to strengthen patients’ ability to reflect upon their thinking, and to recognize and correct their dysfunctional thought patterns and behavior (Moritz et al., 2018; Schneider et al., 2018) in order to ultimately reduce depressive symptoms. Additionally, MCT-Silver aims to increase awareness of depression-related information processing strategies, such as attentional preferences for negative information and mood-congruent memory (Blaney, 1986; Wittekind et al., 2014; McConnell and Troop-Gordon, 2021). Individual modules also address dysfunctional coping strategies (e.g., thought suppression, rumination) and metacognitive beliefs (e.g., rumination helps to solve problems). As a “modern” CBT-intervention, MCT-Silver includes third-wave elements drawn from acceptance and commitment therapy (Hayes et al., 2006) and imagery rescripting (Holmes et al., 2016). The training also addresses age-specific challenges and risk factors for depression in older adults (e.g., loneliness, functional limitations, loss of relationships).

MCT-Silver has not yet been validated for the Portuguese population and studies are needed to prove its efficacy in this population. Therefore, after the completion of a pilot study, a randomized controlled trial will be conducted to evaluate the efficacy of the intervention in older adults with depression. The research question of this study is as follows: “Does MCT-Silver in older adults with depression lead to a greater reduction in depressive symptoms and dysfunctional beliefs as well as improved metacognitive beliefs, quality of life, ruminative responses, self-esteem and attitudes towards aging compared to treatment as usual?.” The pilot study is in preparation. After completion of the pilot study and application of results as necessary (e.g., adjustment of MCT-Silver for the Portuguese population), we will continue with the intervention in order to finalise the RCT.

## 2. Materials and methods

### 2.1. Aim

The aim of this study is to evaluate the efficacy of MCT-Silver and its effects on depressive symptoms, dysfunctional beliefs, metacognitive beliefs, quality of life, ruminative responses, self-esteem and attitudes towards aging. The hypotheses that we want toexamine in this trial are the following:

1. Primary outcome: There will be a greater reduction in self-reported depression (as measured by the Beck Depression Inventory-II) for MCT-Silver vs. TAU (pre-post; pre-follow-up).
2. Secondary outcomes: There will be a greater reduction in clinician-rated depression (MADRS), metacognitive beliefs (Metacognitions Questionnaire-30), self-reported depression (PHQ-9), dysfunctional beliefs (DAS-18B), ruminative responses (RRS), self-esteem (Self-Esteem Questionnaire), quality of life (WHO QOL-Bref??), and attitudes to aging (Attitudes to Aging Questionnaire) for MCT-Silver vs. TAU (pre-post; pre-follow-up).
3. Tertiary outcome: Participants will rate the intervention positively on a satisfaction questionnaire administered after the 8-week intervention period.

### 2.2. Design/methodology

#### 2.2.1. Study design

This study represents an observer-blind, parallel-groups randomized controlled trial.

All procedures follow CONSORT (Consolidated Standards of Reporting Trials) guidelines and follow four phases: enrollment, intervention allocation, follow-up, and data analysis. The follow-up will occur 3 months after completion of the intervention. The trial has been registered at ClinicalTrials.gov (NCT05640492).

#### 2.2.2. Participants

The sample will be selected by a probabilistic method and participants will be randomly assigned to one of two groups (MCT-Silver vs. TAU) based on a randomized plan.

##### 2.2.2.1. Inclusion criteria

(1) Age 60 years and over; (2) score greater than 8 on the Montgomery and Åsberg Depression Rating Scale (MADRS) (which corresponds to mild depression, optimal cut-off) (Zimmerman et al., 2004); (3) ability to give informed consent; (4) sufficient command of the Portuguese language; and (5) willingness to participate in the intervention over the period of the study. The diagnosis of depression will be identified by a trained professional, using the DSM-5 as a reference (American Psychiatric Association, 2014).

##### 2.2.2.2. Exclusion criteria

(1) Lifetime psychotic symptoms (i.e., delusions, hallucinations); (2) current or a history of mania; (3) acute suicidality; and (4) dementia or other neurological disorder which could be related to the onset of depressive symptoms (e.g., Parkinson’s disease, stroke, multiple sclerosis); (5) current substance dependence (substance abuse will be tolerated); (6) not having attended a similar training in the last 3 months; (7) patients with the MMSE score below 24 points will not be included.

Participants will be informed about the study and conditions of participation individually and written informed consent will be obtained from each participant. Participants will be asked if they have a guardian for medical decision-making. In this case, if the potential participant generally meets the criteria for inclusion, a permission to participate will be obtained from the guardian.

#### 2.2.3. Randomization and blinding

**Figure 1:**
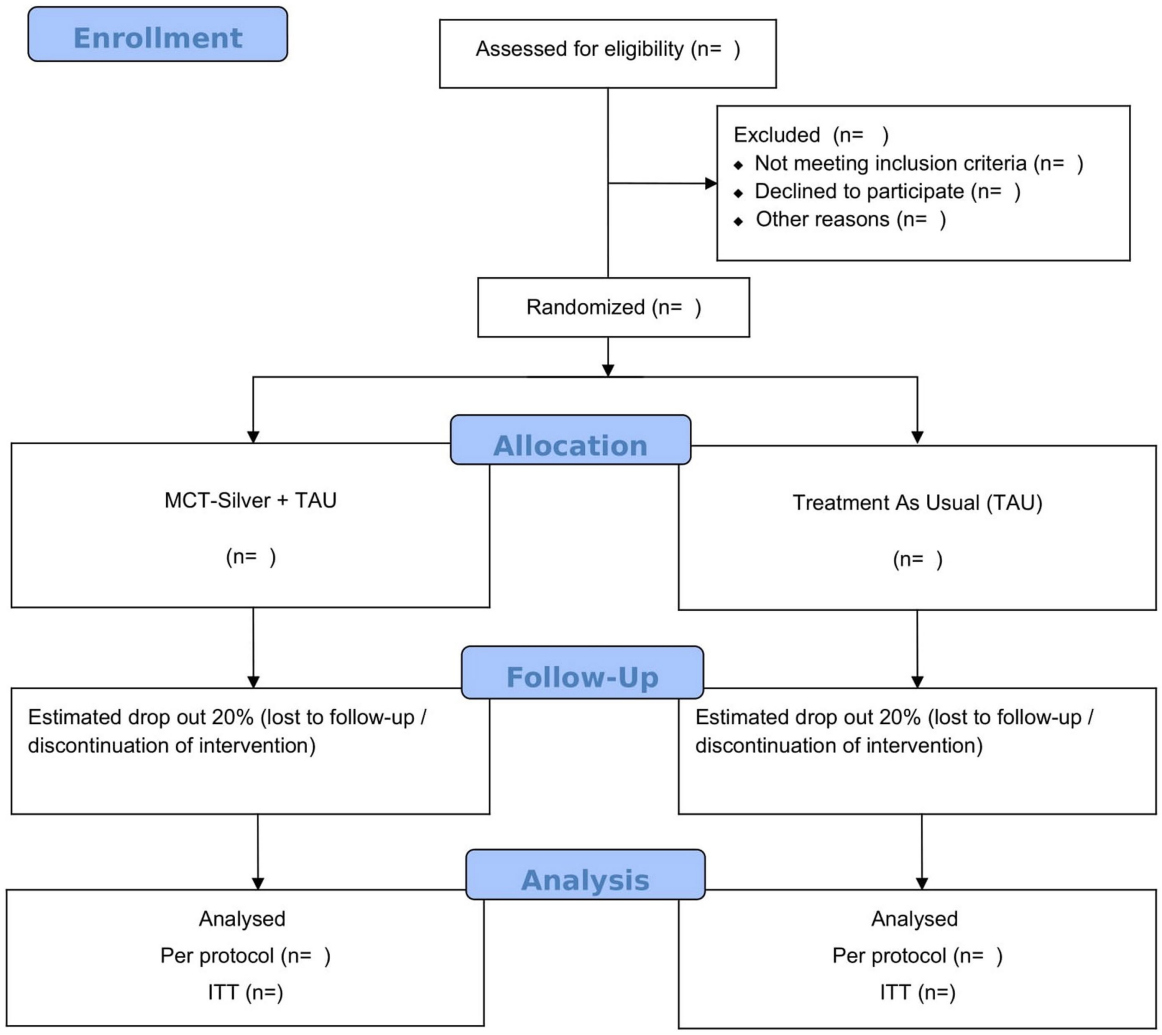
CONSORT flow diagram.

Eligible participants will be recruited at each institution (the mental health service of each hospital that agreed to take part in the study in Portugal) through telephone contact by the researchers in collaboration with the multidisciplinary team. The study will also be publicized in the media so that older adults can contact the researchers if they are interested in participating. A baseline assessment will be carried out and all instruments will be applied after informed consent from participants. All participants who agree to participate in the study will be assigned a code and will be randomly assigned to either MCT-Silver (experimental group where the intervention will be applied) or the control group (no stratification factors). This randomization will be performed using a computer program. The control group will not be administered the intervention (MCT-Silver). In both groups, treatment will be maintained as usual (Treatment As Usual – TAU) and participation in other interventions as well as use of medications will be carefully monitored. Study participants will be informed that they should not reveal which group they are in and will be reminded of this at each testing session. All participants will be re-assessed at the end of the intervention and 3 months after completion, with the application of all planned instruments. Assessments will be performed by the same rater before and after the intervention and at follow-up. Assessors will be trained in the administration of all instruments prior to the start of the study. A third researcher will inform patients of group assignment so that raters will remain blinded throughout the study. The Side Effects of Intervention questionnaire and patient satisfaction with MCT-Silver will be applied to the experimental group post-intervention (Table 1; Figure 1).

**Table 1:** Study schedule.

	Study period				
	Enrolment	Randomization	Intervention (8 weekly sessions)	Post (8 weeks)	Follow-up (3 months)
TIMEPOINT	T-1	T0		T1	T2
Enrolment:					
*Eligibility screen*	X				
*Informed consent*		X			
*Randomization*		X			
Interventions:					
*Treatment As Usual (TAU)*	X	X	X	X	X
*MCT-Silver*			X		
Assessments: *Baseline*		X			
*Post-intevention*				X	
*Follow up*					X

#### 2.2.4. Sample size calculation

In RCTs to calculate the sample size, four factors are required: significance level, power analysis, difference between groups, and standard deviation (SD; Charles et al., 2009). As there is only one RCT in Germany that applied the MCT-Silver, we chose to conduct a pilot study, and the sample size calculation for the RCT is calculated *a posteriori*. For the pilot study, we will include 32 participants, as 15–20 participants are needed to ensure the scientific validity of the results of a pilot study (Hertzog, 2008). The results of the pilot study will provide information on the above four factors as they are necessary for the calculation of the sample size.

#### 2.2.5. Study intervention

##### 2.2.5.1. Treatment as usual

All participants (experimental and control group) will continue to receive treatment as usual (TAU) at their institution. TAU may consist of psychiatric and psychosocial treatment by a multidisciplinary team, which includes mental health psychiatric nurses, psychiatrists, psychologists, social workers, occupational therapists and other mental health professionals. TAU may also include treatment in community settings, day hospital and inpatient settings, as well as antidepressant medication, psychosocial rehabilitation, socialisation groups and psychoeducational and therapeutic groups. The care of participants in TAU will not be limited or influenced in any way. To control for TAU, we will carefully record participation in concurrent treatment. The control group will not participate in the MCT-Silver programme.

##### 2.2.5.2. Metacognitive training for depression (MCT-Silver)

Metacognitive training (MCT) has its origins in 2003 with the development of “metacognitive training for psychosis” (Moritz et al., 2013, 2017). MCT has since been developed for other psychiatric disorders, e.g., depression (D-MCT; Moritz et al., 2013; Jelinek et al., 2015), and an adaptation of the D-MCT, called MCT-Silver (pilot study), has been developed for older adults with depression (Schneider et al., 2018). Although certain terms have been adapted for the Portuguese translation, case examples, exercises and general content were maintained to ensure consistency across countries. However, the intervention and examples can be tailored by the trainers to meet specific group needs of the group concerned (e.g., to reduce the length of sessions or tailor content to specific issues of group members). All information about this programme is available^2^ in English, German and Portuguese.

MCT-Silver is a manualized treatment program comprised of eight modules addressing common cognitive distortions and biases in information processing in depression as well as depression-related behaviors and metacognitive beliefs. Differing from D-MCT, MCT-Silver also includes components drawn from acceptance and commitment therapy as well as imagery rescripting. All content is supported by significant empirical work linking these processes to depression. The topics of MCT-Silver include the following: Modul 1: Mental filter; Module 2: Mood-congruent memory / false memories (Mathews and Macleod, 2005; Moritz et al., 2008); Module 3: “Should” statements (Egan et al., 2011; Mcgrath et al., 2012) and disqualifying the positive (Cane and Gotlib, 1985; Elliott et al., 1997) as well as acceptance of negative feelings (Hayes et al., 1996; Butler and Ciarrochi, 2007); Module 4: Values (Isaacowitz and Seligman, 2002; Hayes et al., 2006; Wrosch et al., 2013); Module 5: Exaggeration/Minimization (Garber and Hollon, 1980; Hoehn-Hyde et al., 1982; Cane and Gotlib, 1985; Wenzlaffand and Grozier, 1988) as well as Attribution Style (Carver and Ganellen, 1983; Wenzlaffand and Grozier, 1988); Module 6: Rumination and Withdrawal (Rood et al., 2009; Seidel et al., 2010; Wells, 2011); Module 7: Jumping to Conclusions (Strunk et al., 2006; Miranda et al., 2008), and Module 8: Self-Worth in Later Life (Davey et al., 2004; Orth et al., 2009; Holmes et al., 2016; Table 2).

**Table 2:** Descriptions of the content of MCT-Silver modules.

Module	Name	Cognitive bias/Behavior	Aims	Training example
1	Thinking and reasoning I	Mental filter, overgeneralization	Identify and modify selective perception (i.e., mental filter) and exaggerated generalizations of negative experiences.	*Modification of overgeneralization of negative experiences*: Situational examples are presented along with possible (depressive) interpretations. More helpful interpretations of these situations are identified.
2	Memory	Mood-congruent memory, false memories	Normalize and explain concentration and memory issue. Increase awareness of memory biases.	*False memory effect*: Participants are asked to remember objects presented in a picture. They are then provided with a list of words and are asked to identify the incorrect objects, which were not presented.
3	Thinking and reasoning II	“Should” statements, “all or nothing” thinking, acceptance of negative feelings related to life changes.	Encourage participants to question rigid and perfectionistic behavior Present the concept of acceptance for negative feelings.	*Examining perfectionistic standards*: The pros and cons of holding oneself to certain (high) standards (e.g., to always have a perfectly clean home) are discussed. *Acceptance*: Identification of areas in life in which participants would like to practice more acceptance.
4	Values	Identifying values and strategies for living a value-based life	Identify personal values.	Values: Communication regarding the importance of improving insight into personal values. Processing of strategies that can support a value-oriented life.
5	Thinking and reasoning III	Magnification and minimization, depressive attributional style	Identify and modify biases in judging the extent and consequences of perceived successes and failures.	*One-sided attributions*: Examples of one-sided attributions are presented, and participants are encouraged to identify multiple causes for an outcome.
6	Behaviors and strategies	Dysfunctional coping strategies: withdrawal, rumination, thought suppression	Reduce dysfunctional behaviors associated with depression. Develope new helpful coping behaviors.	*Helpful coping strategies*: Mindfulness exercise to practice gaining inner (psychological) distance.
7	Thinking and reasoning IV	Jumping to conclusions, mind reading, fortune telling (catastrophizing)	Identify instances of jumping to conclusions. Encourage consideration of multiple sources of information before reaching a conclusion.	*Considering alternative information*: Paintings are presented and participants are asked to guess the correct title from a list of choices.
8	Self-esteem	Self-esteem, changing negative self-perceptions through imagery	Communicate strategies to improve self-esteem Reduce and modify unfair comparisons (e.g., with the younger self).	Imagination exercise: Transformation of negative images into strong, positive images (e.g., transformation of a weak, nervous chick into a proud eagle).

**Figure 2:**
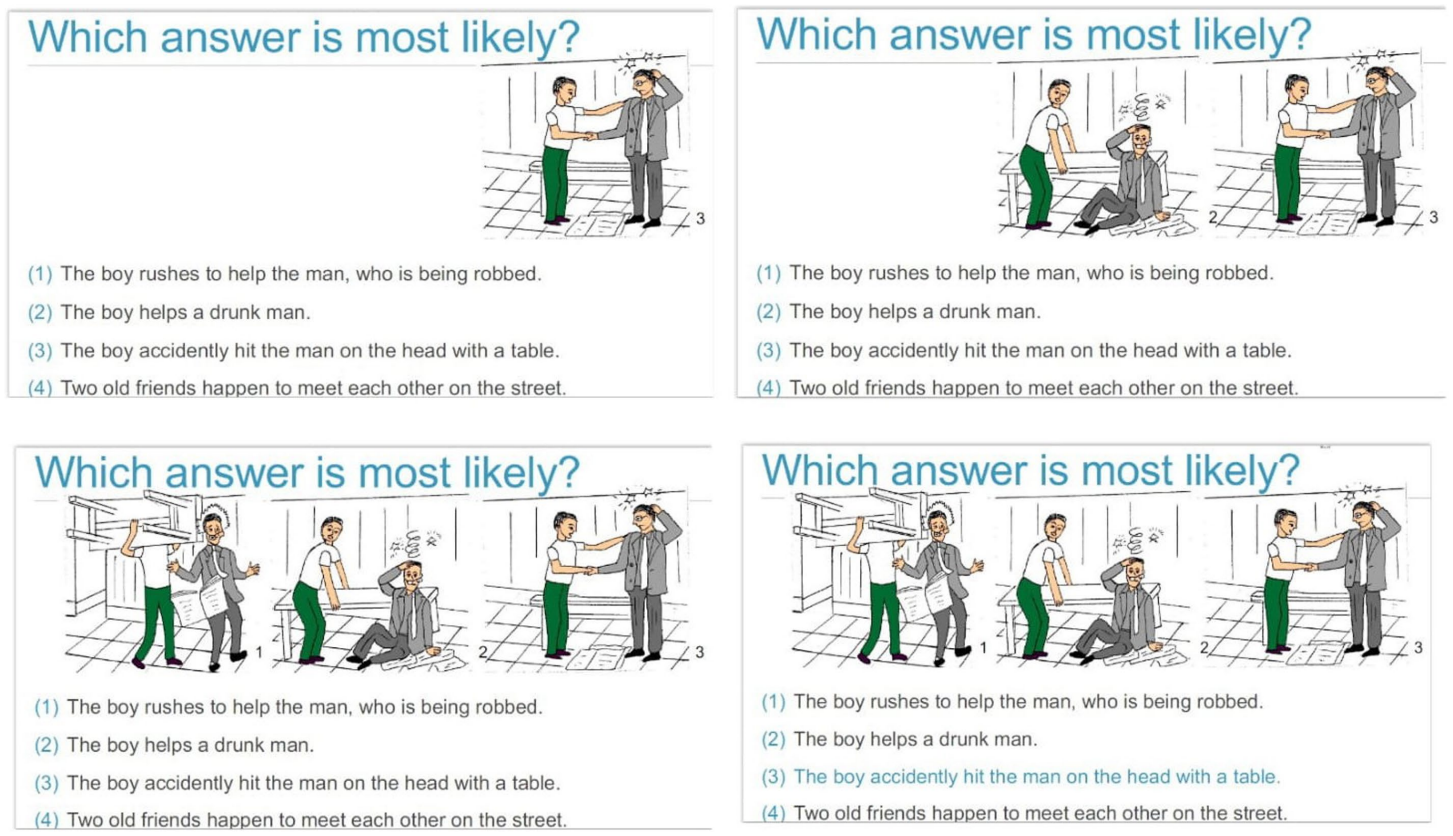
Example of Module 7: Jumping to Conclusions.

Figure 2 shows an example of Module 7: Jumping to Conclusions.

Through interactive exercises and a structured multimedia presentation, MCT-Silver aims to convey knowledge about cognitive distortions, helps participants to reflect critically about and change the content of their thoughts, and acquire new strategies to solve problems. Each session lasts 45–60 min (Jelinek et al., 2022).

In the current study, MCT-Silver will be applied to the experimental group by mental health and psychiatry professionals with experience in the care of individuals with depression. A training course on the application of the MCT-Silver programme will be completed by these professionals prior to the start of the study. Administration of the training will also be monitored and supervised by the study PI (LGP). For adherence monitoring of the MCT-Silver application the sessions will be recorded, and no images of the participants will be taken to safeguard ethical issues.

MCT-Silver sessions will be applied once a week for a total of eight sessions. The intervention will be applied face-to-face in groups in a quiet room of the institution to which the group belongs.

#### 2.2.6. Data collection and outcome measures

The instruments will be administered to all study participants through an interview at three planned assessments (Table 3). To characterize the sample, sociodemographic, and clinical data will be collected [age, sex, marital status, cohabitation, educational level, professional/employment status, duration of depression diagnosis (if applicable), number of psychiatric hospitalizations, medication, type of treatment, and substance use; Table 4]. These data will be collected before the start of the MCT-Silver intervention for all participants.

**Table 3:** List of study measures and questionnaires.

Instruments	Baseline	8 weeks (post)	3 months (follow-up)
Mini Mental State Examination	X		
Beck’s Depression Inventory (BDI-II)	X	X	X
Patient Health Questionnaire (PHQ-9)	X	X	X
Montgomery-Asberg Depression Rating Scale (MADRS)	X	X	X
Metacognitions Questionnaire (MCQ-30)	X	X	X
Dysfunctional Attitudes Scale (DAS-18B)	X	X	X
WHOQOL-BREF Item 1	X	X	X
Ruminative Response Scale (ERR-10-A)	X	X	X
Rosenberg Self-Esteem Scale	X	X	X
The Attitudes to Ageing Questionnaire (AAQ-12)	X	X	X
Intervention questionnaire and patient satisfaction		X	X

**Table 4:** Summary of the clinical and demographic characteristics of the sample.

Questions	Answers						
Age							
Gender	Female	Male					
Marital status	Single	Married/ marital partnership	Widowed	Divorced/separated			
Level of education	Cannot read or write	Can read and write, but has not attended school	Attended school, but not higher education	Number of years of schooling	Higher education		
Who you live with	Institution	Alone	Spouse/partner	Child	Brother/sister	Other relative	Other non-family member
Labour status	Employed	Unemployed	Retired	Retired on invalidity	On sick leave	Current or past occupation	
Do you take any medication? If yes, which one?	No	Yes	If so, which one?				
Have you ever been admitted to a psychiatric ward?	No	Yes	How many times?				
Have you ever been to a psychiatric day hospital?	No	Yes	How many times?				
Are you followed up in follow-up appointments/ programmes?	No	Yes	Which ones?				
What is your current substance consumption?	None	Tobacco	Alcohol (more than 3 drinks a day)	Other drugs, which ones?			
Do you have any diagnosed illness(es)?	No	Yes	If so, which one or ones?				

##### 2.2.6.1. Mini Mental State Examination (MMSE)

The MMSE will be applied to screen for cognitive deficits. Participants with cognitive impairments according to the cut-offs for the MMSE will be excluded from the study. This instrument evaluates cognitive function and was developed by Folstein et al. (1975). It is composed of six groups of questions that assess orientation to time and place, attention / concentration, short-term memory (recall), language skills, visuospatial abilities and ability to understand and follow instructions. Higher scores indicate better cognitive ability. The MMSE was adapted for the Portuguese population (Guerreiro et al., 1994). The cut-off for establishing cognitive impairment adjusted for years of education and, thus, also exclusion from the study is as follows: 0 years education ≤15 points; 1-11 years of education ≤22 points; 11+ years of education ≤24 (Folstein et al., 1975).

#### 2.2.7. Primary outcome measure

##### 2.2.7.1. Beck’s Depression Inventory (BDI-II)

The scale developed by Beck et al. (1961) was adapted and validated in Portuguese by Martins et al. (Martins et al., 2000; Coelho et al., 2002) aims to differentiate between depressed and non-depressed individuals, as well as to measure the severity of depressive symptomatology. It includes 21 items grouped into three factors: Cognitive Factor (*n* = 8 items); Affective Factor (*n* = 6 items); and Somatic Factor (*n* = 7 items). Symptom severity over the past 2 weeks is ranked on a four-point Likert scale, (e.g., sadness, 0 – I do not feel sad; 3 – I am so sad or unhappy that I cannot take it anymore) (Araújo, 2011). The BDI-II has adequate internal consistency, considered by some authors as “excellent” (Coelho et al., 2002). Scores ≤13 indicate minimal depressive symptomatology; scores between 14 and 19 indicate mild depression; scores between 20 and 28 indicate moderate depression; and scores >28 indicate severe depression (Beck et al., 1966).

#### 2.2.8. Secondary outcome measures

##### 2.2.8.1. Montgomery-Asberg Depression Rating Scale (MADRS)

This instrument is a 10-item rating scale developed by Montgomery and Åsberg (1979). The severity of each symptom is rated from 0 to 6, with higher scores indicating more severe symptoms. Snaith et al. (1986) proposed the following total score interpretations: 0 –6 indicate no symptoms; 7 – 19 indicate mild depression; 20 – 34 indicate moderate depression and 35 and greater indicates severe depression. The reliability of the MADRS ranges from α = 0.64 – 0.89 (Huijbrechts et al., 1999). A methodological study to adapt this instrument to the Portuguese population will be conducted within this study.

##### 2.2.8.2. Patient Health Questionnaire (PHQ-9)

The PHQ-9 was developed by Kroenke et al. (2001) and has been adapted and validated for the Portuguese population (Monteiro et al., 2013). The Portuguese version of the PHQ-9 has satisfactory internal consistency (Cronbach’s alpha = 0.86) and showed strong convergent validity with the BDI (*r* = 0.85; *p* < 0.01) (Monteiro et al., 2013). The PHQ-9 consists of nine items that assess the severity of depression-related symptomatology on a 4-point Likert scale (from 0 “Never” to 3 “Nearly every day”). Summed scores range from 0 to 27. Higher scores indicate more severe depression: scores between 0 and 4 indicate minimal depression, scores between 5 and 9 indicate mild depression, scores between 10 and 14 indicate moderate depression, scores between 15 and 19 indicate moderately severe depression, and scores between 20 and 27 indicate severe depression (Kroenke et al., 2001).

##### 2.2.8.3. Metacognitions Questionnaire (MCQ-30)

The MCQ-30 was developed by Wells and Cartwright-Hatton (2004) and is available in Portugese (Dinis and Gouveia, 2011). The questionnaire assesses metacognitive beliefs on five subscales: cognitive confidence, positive beliefs about worry, cognitive self-consciousness, negative beliefs about the uncontrollability of thoughts and danger, and beliefs about the need to control thoughts. The Portuguese version of the MCQ-30 has good internal consistency (Cronbach’s alpha = 0.91; Dinis and Gouveia, 2011). In our study we will apply three subscales of MCQ-30: (1) Positive Beliefs about worry (6 items); (2) Negative Beliefs about thoughts concerning uncontrollability and danger (6 items), and (3) beliefs about the need to control thoughts (6 items). We will only use these three subscales because they are most closely related to depression (Ruiz and Odriozola-González, 2015) and because the remaining two subscales of the MCQ-30, “cognitive confidence” and “cognitive self-awareness,” do not correspond to the concepts of MCT (Jelinek et al., 2013, 2017).

##### 2.2.8.4. Dysfunctional Attitudes Scale (DAS-18B)

The DAS-18B is a self-report questionnaire designed to assess dysfunctional attitudes, thoughts, and schemas associated with depression. It consists of 18 items answered on a seven-point Likert scale (1 = total agreement to 7 = total disagreement). Higher scores indicate more dysfunctional attitudes (Rojas et al., 2015). A methodological study to adapt this instrument to the Portuguese population in an 18-item version will be carried out within the scope of this study.

##### 2.2.8.5. WHOQOL-BREF item 1

The global item (“How would you rate your quality of life?”) of the WHOQOL-Bref (World Health Organization Quality of Life Instruments - Bref) will be used for this study. The WHOQOL-Bref was developed by the WHO in 1998 (The Whoqol Group, 1998) and adapted for the Portuguese population by Vaz Serra et al. (2006).

##### 2.2.8.6. Ruminative Response Scale (ERR-10-A)

This scale is the reduced version (10 items; Treynor et al., 2003) of the Response Styles Questionnaire (RSQ: Response Styles Questionnaire; Nolen-Hoeksema and Morrow, 1991), which was adapted and validated for the Portuguese population (Dinis et al., 2011). This instrument assesses ruminative responses. The ERR-10-A includes 10 items with two factors. The “reflection” factor refers to attempts to understand the reasons for depressed mood (Cronbach’s alpha = 0.75). The “brooding” factor refers to perseverative thinking focused on negative consequences of depressed mood and obstacles to problem-solving (Cronbach’s alpha = 0.76). Respondents are provided with a series of statements referring to “what they usually do when they feel sad depressed or down” and provide answers on a four-point Likert scale, ranging from 1 (‘hardly ever’) to 4 (“almost always”; Dinis et al., 2011).

##### 2.2.8.7. Rosenberg Self-Esteem Scale (RSE)

The Rosenberg Self-Esteem Scale (Rosenberg, 1965) is available in Portuguese (Cronbach’s alpha = 0.86; Santos and Maia, 2003) and is comprised of 10 items concerning self-confidence and self-depreciation. A high score indicates positive feelings that the individual has about himself, leading to self-respect and the awareness that he is capable, without the feeling of superiority. A low score expresses low self-esteem, which implies self-rejection, dissatisfaction, and contempt for oneself (Santos and Maia, 2003).

##### 2.2.8.8. The Attitudes to Ageing Questionnaire – Short form (AAQ-SF)

This instrument was originally created in its 24-item form by Laidlaw et al. (2007) and a short-form (AAQ-SF) was later developed (Laidlaw et al., 2018). The AAQ-SF is a 12-item rating scale and utilizes a 5-point Likert scale. Items query participants’ attitudes regarding aging on three factors (psychosocial loss, psychological growth, physical change). The 12-item AAQ-SF showed adequate internal consistency and confirmatory factor analysis confirmed that the structure of the AAQ-SF reflects that of the original 24-item AAQ (Laidlaw et al., 2018).

##### 2.2.8.9. Intervention questionnaire and patient satisfaction

Assessment of satisfaction with care/treatment should be evaluated in patient-centered approaches (Pinho et al., 2021). The Side Effects of Intervention questionnaire and patient satisfaction with MCT-Silver will be applied to the experimental group post-intervention. To assess the participants’ acceptance of the interventions, a self-designed subjective evaluation scale will be used, consisting of 21 items. This includes 18 items (e.g., “I found the Metacognitive Training programme useful and important”; “I able to cope better with my illness after completing the training”; “The training programme helped me to gain a better understanding of my illness.”) in which participants are asked to respond to the items on a 5-point Likert scale (1 = totally agree; 2 = agree; 3 = neutral; 4 = disagree; 5 = totally disagree). For the interpretation of the evaluation ratings, both positive ratings (i.e., totally agree and agree) are combined for the positively worded items and vice versa for the reversed items. There are also three open-ended items (e.g., “I particularly liked:…”). This information will be useful to understand patient acceptance of and satisfaction with the participants in the study.

#### 2.2.9. Data analysis

The data will be analysed using Statistical Package for Social Sciences software (SPSS®) version 24.0 for Windows. Descriptive analysis will be used to characterize the sample. Within-group differences (t0 to t1 and t0 to t2) will be examined using paired sample *t*-tests. To evaluate change in primary and secondary outcomes between the two groups, we will perform ANCOVAs with treatment as the between-subject factor (MCT-Silver + TAU vs. TAU), the difference score of the outcomes (t1 – t0 and t2 – t0, respectively) as the dependent variable, and the baseline score of the respective outcome as the covariate. We chose ANCOVAs with difference scores instead of a repeated measures ANOVA to avoid regression to the mean and to control for potential baseline differences.

Intention-to-treat (ITT) analyses (including all participants who provided baseline data) using multiple imputation (MI) will be conducted. Analyses utilizing a complete cases sample (CC; including only complete data—i.e., baseline, post, and follow-up) will also be conducted. We will use analogous models to assess changes (t0-t1; t0-t2) for the secondary outcomes (WHOQOL-BREF item 1, DAS-18B, ERR-10-A, AAQ12, MCQ-30, MADRS, PHQ-9, RSE). We will also conduct planned moderation analyses for the primary outcome at post-assessment (BDI-II). Demographic characteristics (age, sex, education) and clinical characteristics (previous or current treatment, comorbid anxiety disorder, number of sessions attended) as well as baseline scores on secondary outcomes (WHOQOL-BREF item 1, DAS-18B, ERR-10-A, AAQ12, MCQ-30, MADRS, BDI, RSE) will be examined separately using moderation models as defined as defined by the PROCESS SPSS macro (model 1) [59]. Self-rated depression difference scores (t0–t1 and t0–t2) will be entered as the dependent variable (Y) and depression severity at t0 as the covariate.

### 2.3. Ethical considerations and dissemination

Approval will be obtained from the ethics committees of all institutions where the study will be carried out. All participants will be informed of the study’s objectives, methodology, benefits, and possible risks. All participants will sign a written informed consent expressing their agreement to participate in this study. Participants’ confidentiality will be ensured during all study procedures. Participants will be informed that they may withdraw their participation at any time without penalty or other consequence. Only research team members and healthcare professionals who care for participants have access to participant data. This data will be destroyed 5 years at the end of the study. If there is a need to change planned procedures, and that may have an impact on the execution of the study (e.g., changes in the study design, in the study objectives and in the study procedures), they will be reviewed by independent investigators and communicated to the ethics committees of all institutions where the study was carried out. The results of the study will be disseminated through oral communications and posters at conferences, and publications in scientific journals.

### 2.4. Validity and reliability/rigour

Variables such as educational level, duration of mental disorder, and type of treatment may influence the results of this study. The proposal to use the stratified random sampling method in this study aims to minimize confounding bias. All procedures, including the implementation of the intervention will be carried out after trainers have completed MCT-Silver training to minimize the risk of bias. Participants will be randomly allocated to either the experimental group [MCT-Silver + Treatment As Usual (TAU)] or the control group (TAU only). A computer-generated stratified random sampling method will be applied taking into consideration an equitable distribution by sex and age to promote sex and age equality and inclusiveness. Finally, all raters will be blinded to participant group assignment.

## 3. Discussion

MCT-Silver is a low intensity, CBT-based group intervention, which appears to have great potential in reducing depressive symptoms but further studies are needed to better validate its efficacy as the pilot study was limited due to lack of a control group (Schneider et al., 2018) as well as the lack of follow-up assessments. Only one RCT on MCT-Silver has been conducted (in Germany) (Schneider et al., under review).^3^ The efficacy of MCT-Silver for older adults with depression has not yet been studied in the Portuguese population and, therefore, the development of the present trial is essential.

Portugal is one of the countries of the Organization for Economic Co-operation and Development (OECD) with the highest consumption of antidepressants (OECD, 2019) and, despite international guidelines recommending complementary treatment with non-pharmacological interventions (American Psychological Association, 2019), in Portugal this practice is not common. Most people with depression are only treated with medications.

It is expected that the results of this trial will provide support for the efficacy of the Portuguese version of MCT-Silver on the reduction of depressive symptoms. Moreover, it is anticipated that participants in the MCT-Silver group will demonstrate greater reductions in metacognitive beliefs, dysfunctional attitudes, ruminative responses and (negative) attitudes towards aging, as well as greater improvements in quality of life and self-esteem compared to a TAU group. The results of this trial are expected to allow future implementation of MCT-Silver by mental health and psychiatry professionals in various contexts. The MCT-Silver programme aims to contribute to the psychosocial rehabilitation of older adults with depression. The trial illustrated in this protocol intends to assess the efficacy of MCT-Silver in the Portuguese population.

Since people with depression often have cognitive deficits (Muhammad and Meher, 2021), patients with significantly impaired cognitive abilities may have difficulty understanding MCT-Silver exercises. To overcome this limitation, we will apply the MMSE to screen for cognitive deficits. Other limitations may be transportation difficulties getting to sessions regularly, as well as health problems, which may limit participation in the study or the changes in medication during the course of the study.

Minimal negative side effects of D-MCT have been previously reported (Dietrichkeit et al., 2021). We anticipate similar findings, such as disappointment that the training did not result in (greater) symptom improvement and being more burdened by symptoms because patients may think about them more during the training. Attendance (i.e., coming to appointments) may also be perceived as stressful. It is also possible that patients could experience a clinically significance worsening of symptoms. In this case, participation in the study would be discontinued and, if necessary, participants would be offered assistance in identifying needed treatment.

This study includes many measurement instruments and were chosen to improve comparability with previous trails on MCT-Silver and D-MCT (see Jelinek et al., 2015) (Schneider et al., under review, see footnote 3). Nonetheless, completion of study measures can be burdensome and time-intensive for participants. Given that this is the first MCT-Silver trial in Portugal and participants are receiving a psychological intervention, we believe that the potential knowledge to be obtained from the study justifies participants’ time and potential burden related to participation in the study. Additionally, participants are allowed to discontinue testing and will not be penalized for this. Use of self-report measures is common in RCTs of psychological interventions and provide indications regarding patients’ perceptions of symptoms and suffering from these symptoms. Nonetheless, responses are impacted by many factors, including social desirability and insight, as well as reading competence and motivation, which may limit the validity of our findings. For this reason, we have also included a clinician-rated measure of depression (MADRS). The results of the RCT will be implemented to improve the training, provide information regarding efficacy among Portuguese patients (vs. TAU) and, in the case of efficacy, to support dissemination and uptake in Portugal, which would lead to a reduced treatment gap among older adults with depression.

## Author contributions

LP and BS initiated the study design. LJ, CF, ML, and SM reviewed the study design. CS, LP, BM, and BS wrote the first draft of the manuscript. LP, BS, LJ, and SM provided theoretical, practical, and research expertise on metacognitive training. CF, ML, LJ, and SM revised the manuscript. All authors contributed to the refinement of the study protocol and approved the final manuscript.

## Footnotes

^1^www.uke.de/mct-silver

^2^http://www.uke.de/mct-silver

^3^Schneider, Brooke C., Veckenstedt, Rute, Karamatskos, Evangelos, Ahlf-Schumacher, Jana, et al. (under review) "Efficacy and moderators of metacognitive training for depression in later life (MCT-silver): a randomized controlled trial."
